# Surgical VLS Therapy of Oesophageal Achalasia in Pediatric Age: Four Case Reports

**Published:** 2020-05-31

**Authors:** A. Garzi, M. Prestipino, M.S. Rubino, E. Calabrò

**Affiliations:** 1 Division of Pediatric M.I.S. and Robotic Surgery University of Salerno, Italy; 2 Division of Pediatric Surgery A.O. S. Maria della Misericordia Perugia, Italy

**Keywords:** achalasia, pediatric age, laparoscopic approach

## Abstract

The Authors present a retrospective review of their record of cases, characterized by 4 cases of achalasia in which it was performed a Heller myotomy with front fundoplication (Thall) in laparoscopic approach in the period from 2012 to 2019.

In paediatric achalasia, the laparoscopic Heller myotomy seems to be the best treatment because of its multiple advantages offered by the minimally invasive technique. First of all, thanks to the video-technique, which allows a complete and extended myotomy, the accuracy of this operation is maximized; moreover, the post-operative pain is widely reduced, thanks to the minimal dissection and traction of the tissues; finally, but not negligible, this approach ensures a better aesthetic result than the classic open technique. With regard to the front fundoplication, the Authors suggest that it is mandatory because, even if it extends the operating time, it ensures a natural protection to the myotomy herniated mucosa and avoids gastro-oesophageal reflux, which often occurs after the surgical correction, thus obliging to perform a reoperation.

## I. INTRODUCTION

Described for the first time in 1694 by Willis, Esophageal achalasia, represents an idiopathic motor impairment of the smooth muscle component of the esophagus, characterized by a failure of post-swallowing relaxation of the lower esophageal sphincter (LES), which remains closed, with the replacement of the physiological peristalsis with simultaneous, repetitive and propulsive contractions (tertiary contractions).

A progressive dilatation upstream of the achalasic segment (megaesophagus), is caused by the stagnation of solid and liquid foods inside esophagus; in fact, only when the hydrostatic pressure of the esophageal content is able to overcome the functional obstruction, the passage of the food bolus into the stomach occurs (1).

Although it is the most known and most common disorder in primary and specific alterations of esophageal motility, with an incidence of 1 case/100000 per year, in a population between 30 and 50 years of age and with a slight prevalence for male sex, achalasia is to be considered a rare disease in childhood (1,6 – 2 % of the total people affected) (2).

About the denervation, the aetiology remains unknown; a genetic predisposition has been hypothesized, thanks to occasional familial aggregations reported in the literature and by the association of the achalasia with the HLA DQW1 phenotype.

Furthermore, the finding of achalasia in children with a Binder syndrome (nasal hypoplasia) as well as the common embryological origin of the nasal gem and myenteric plexus (neural crest) suggested the thesis of a new neurocrestopathy (3).

The physio-pathological mechanism underlying the disease consists of the reduction or absence (agangliosis) of the argyrophilic neurons, which is well shown at the level of the myenteric plexus. The loss of argyrophilic ganglion cells, which generates abnormal movements of the oesophageal musculature above 50%, is progressive: this explains the difference in the intensity of the disease and the clinical feature (4).

During the period from August 2012 to September 2019, 4 young patients with esophageal achalasia were treated with video-laparoscopy approach at the Pediatric Surgery Section of the University of Salerno. The clinical situation, characterized by dysphagia, required diagnostic exams such as endoscopy and upper digestive X-ray with barium, which showed oesophageal achalasia.

The patients were 3 males and 1 female, with an age between 5 and 16 years (mean 10 years) who underwent surgical laparoscopic Heller myotomy with anterior anti-reflux fundoplication. The average time of the surgical operation, entirely executed with laparoscopic technique, was 155 minutes (time-range between 130 and 180 minutes). In 1 case, a perforation of the oesophageal mucosa occurred; it was immediately treated without any complication. The stomach probe was held in place for 24/48 hours and patients were fed on the third post-operative day. The follow-up lasted from a minimum of 18 to a maximum of 60 months and it included a clinical examination and an upper digestive X-ray.

### CASE 1

R. T.:16-year-old female patient with Rett’s syndrome at IV stage. Weight loss and difficulty in eating with frequent regurgitation and episodes of bronchitis ab ingestis had been evident for about three months.

A chest x-ray examination with esophagogram showed a noticeable dilation of the esophagus with slow transit of the contrast and almost no peristalsis; late radiograms demonstrated stagnation of barium in the esophagus. An EGDS examination was then performed, which showed the presence of ectasic and hypomobile esophagus. The cardias appeared spastic, with a “rosette” appearance, and the passage of the probe in the gastric cavity occurs with a sign of the “stream”; lesions of the esophageal and gastric mucosa were not appreciated.

The patient was, therefore, subjected to extra-mucosal cardio-myotomy surgery sec. Heller and antireflux plastic acc. Thall in video-laparoscopy, with gastrostomy packaging sec. Stamm and intraoperative manometric control of LES pressure. At the end of the surgery, she was admitted to the Resuscitation Department for a better monitoring of the cardio-respiratory functional recovery; she was discharged in the sixth post-operative day, after being extubated. She was admitted to the Department of Infant Neuropsychiatry and, after about a week, she suffered from an episode of respiratory arrest with severe bradycardia. Then, she was hospitalized in Resuscitation and intubation and monitoring of cardiorespiratory parameters were performed; a chest x-ray examination had showed the presence of a left pleural effusion with basal dysventilation.

A drainage of the pleural fluid was carried out with a temporary improvement of the respiratory symptoms and reduction of the effusion; the patient was discharged after about a week.

After about a month after surgery, the patient again suffered from respiratory difficulties, hyperpyrexia and somnolence; a chest x-ray examination showed the presence of a left pleural effusion and pulmonary atelettasia. A pleural drainage was positioned, reducing the effusion and resuming the ventilation on the left. In addition, a bronchoscopy was performed which reported the presence, in both bronchial branches, of mucopurulent secretions, which were then aspirated. The cultural examination of the endopleural fluid and urine was positive for Pseudomonas aeruginosa; the patient was, therefore, treated with a specific antibiotic therapy.

For about a week, the patient continued to have breathing difficulties with basal dysventilation, more pronounced on the left, hyperpyrexia and somnolence. For better control of ventilation, tracheostomy was performed in agreement with the parents. Respiratory symptoms and general clinical conditions have been normalized three days after tracheostomy.

After about a week, the gastrostomy was removed and the patient underwent total parenteral therapy via peripheral venous access; a partial digestive x-ray examination showed a normal appearance of the esophagus with peristalsis maintained and, after six days, the feeding was resumed orally. After that, the patient was able to eat regularly.

### CASE 2

A.D.: 5-year-old male patient, born eutocial at term and suffering from spastic tetraparesis, periventricular and cystic leucomalacia from perinatal suffering.

At the age of three he began to report frequent episodes of bronchitis, treated with antibiotic and cortisone therapy. At the age of five he came to our observation for the appearance of dysphagia and weight loss (weight at the entrance: 10.5 Kg). A chest x-ray examination showed a widening of the mediastinum with a right-sided deviation of the trachea and mediastinal vessels, with minimal layers of paramediastinal pneumothorax on the right.

**Fig. 1:**
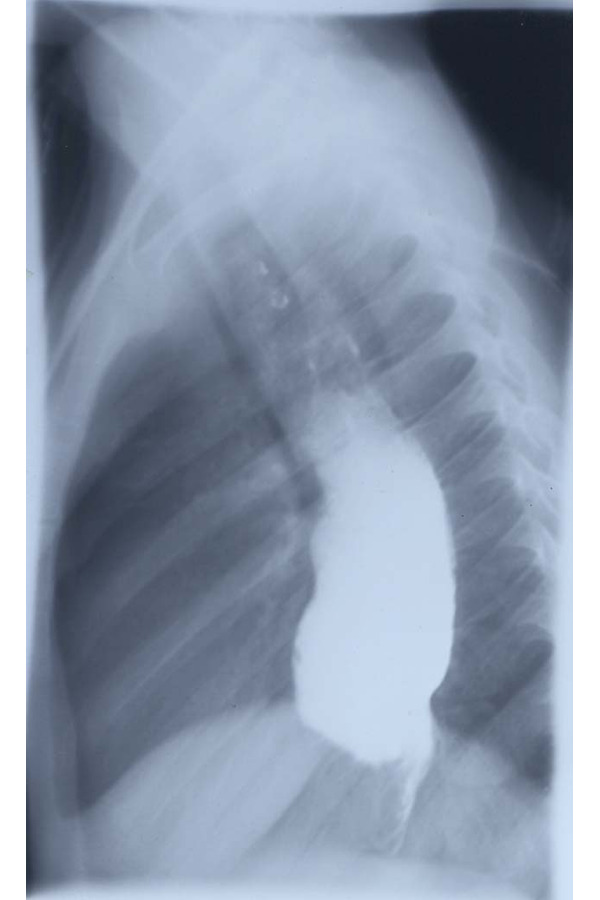
Mouse-tail image of the achalasic segment.

Subsequently, a chest x-ray examination with esophagogram was performed, which showed a remarkable dilation of the esophagus with slowing transit of the contrast and peristalsis almost absent. In addition, there were images of parietal minus at the level of the distal third of the esophagus, which were attributed to alimentary residues; late radiograms demonstrated stagnation of barium in the esophagus (Fig. 1).

An EGDS examination was then performed; this one showed the presence of hypomobile ectasic oesophagus containing moderate amounts of ingestion. The cardias appeared spastic, with aspect to “rosette”, and the passage of the probe in the gastric cavity happened with sign of “stream”; lesions of the esophageal and gastric mucosa were not appreciated. Pneumatic dilations of the lower third of the oesophagus up to 20 mm in diameter were attempted, without tearing the mucosa. The diagnosis of esophageal achalasia was, moreover, confirmed by the manometric investigation.

Pending surgery, the child underwent total parenteral nutritional therapy via a central venous catheter placed in the right subclavian vein. After about a week, the child underwent surgery of cardio-miotomy extramucosa sec. Heller and antireflux plastic sec. Thall in video-laparoscopy.

In the postoperative period, the child suffered from mild microcytic anaemia due to deficiency of iron, which was treated with martial therapy for about a week and he had a positive coprocolture for Candida that was treated with antifungals. Overall, however, there was a rapid resumption of the clinical condition of the child that, from the fifth post-operative day, began able to feed orally with liquid diet that was continued for about a month.

**Fig. 2:**
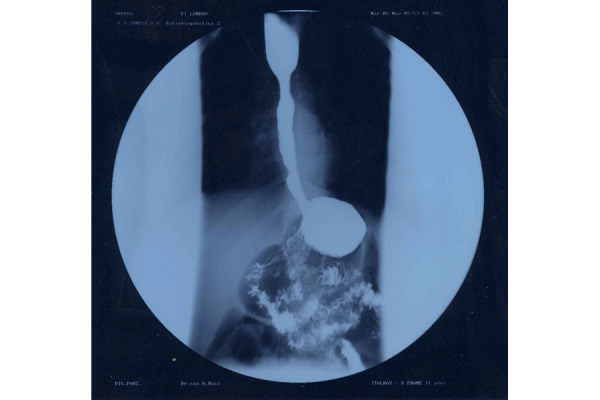
Image of the LES one month after the surgery

A partial digestive x-ray examination, performed one month after surgery, showed a normal appearance of the oesophagus with maintaining peristalsis (Fig. 2).

In the follow up, the child grew well and regularly fed on a free diet.

### CASE 3

N.F.: 8-year-old male patient, born from caesarean section from second pregnancy. Weight at birth of 2500 grams. He was affected by hypokinetic-rigid syndrome associated with tremors mixed at right hemilate with Parkinson’s disease-like symptoms. In the previous months he suffered from frequent regurgitations associated with weight loss. At the time of the hospitalization the patient had a weight of 29 kg.

**Fig. 3:**
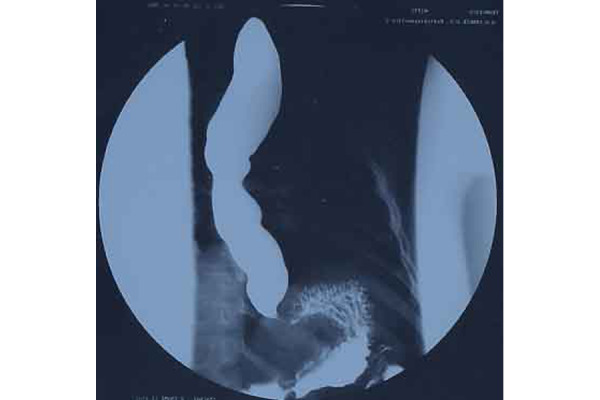
Preoperative image of LES

A chest x-ray with esophagogram was performed (Figure 3), which evidenced a discrete dilation of the esophagus up to the sub-stenotic vestibular tract that allowed small quantities of contrast to pass through. It was decided to execute an EGDS that described aspect of the esophagus compatible with achalasia with minimal notes of esophagitis. The pH-metric trace showed a reduced number of long-lasting gastroesophageal refluxes. The diagnostic conclusion was pH-metric trace compatible with esophageal achalasia.

To confirm the suspicion of achalasia, oesophageal manometry was executed, which identified a lower esophageal sphincter with higher pressure values than normal, with incomplete post-swallowing relaxation. The study of the body revealed the absence of peristalsis with non-propagated tertiary waves of low amplitude with protracted duration. The monometric picture was suggestive for esophageal achalasia.

Therefore, the surgery of cardio-miotomy extramucosa sec. Heller and antireflux plastic sec. Thall in video-laparoscopy was performed, with the placement of nasogastric tube, which was removed on the first post-operative day.

**Fig. 4:**
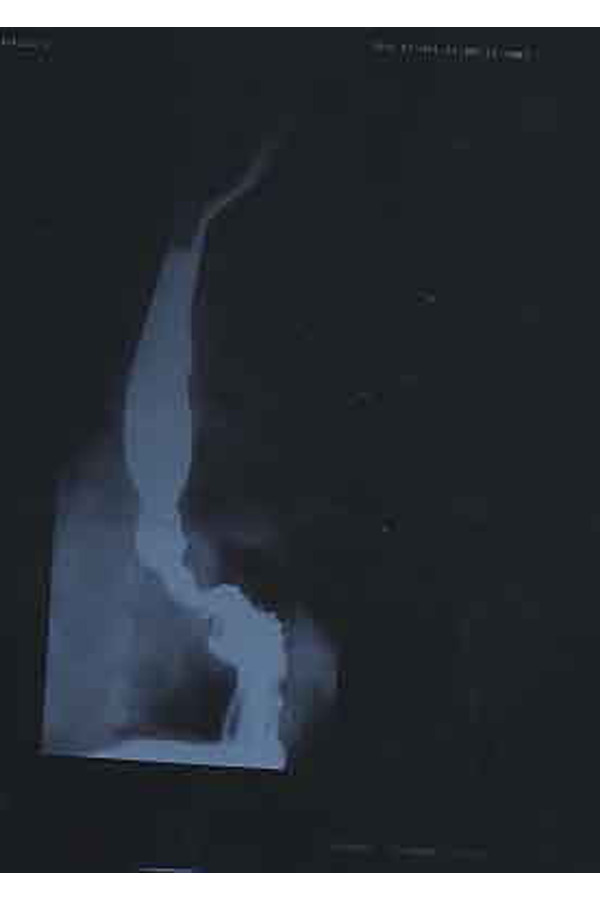
Post-operative image of LES

The postoperative course was normal, with discharge on the seventh day. At the check, three months after surgery, the execution of an upper digestive x-ray (Figure 4) highlighted: “Post-surgery control shows fast transit of the opaque meal with a clear reduction of oesophageal dilation.” There was also a marked increase in weight of 9 kg.

### CASE 4

DM.: 12-year-old male patient, born eutocical at term. For about 5 months he reported dysphagia to both solids and liquids sometimes accompanied by vomiting. He also had halitosis, anorexia and weight loss. At that time the patient weighed 39 kg.

**Fig. 5:**
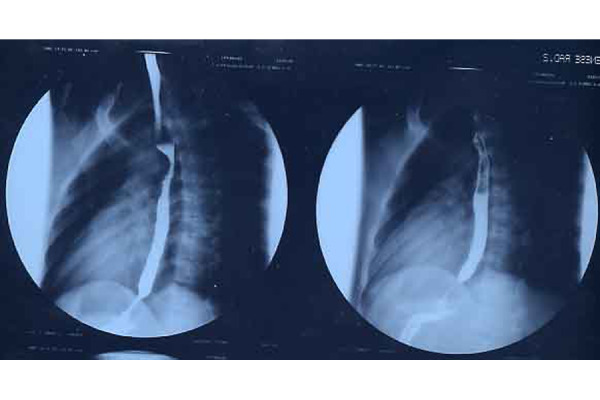
Preoperative image of LES

The following diagnostic tests were performed:

- Double contrast upper digestive x-ray (Figure 5): it showed achalasia with a slowing of the oesophageal transit with moderately increased caliber of esophagus, the contrast formed hydroaero levels in orthostatism caused by concentric stenosis with mouse-tail morphology of the diaphragmatic tract;
-EGDS: it showed slightly ectasic and apparently hypomobile esophagus. The slight reduction of the viscera lumen caliber caused a “spastic” attitude in the terminal section, for the length of about 2 cm, 10 mm close to the Gastro-esophageal junction.
-Oesophageal manometry: the lower esophageal sphincter had upper than normal limit pressure values, with incomplete and occasional post-swallowing relaxation. The peristalsis was present and propagated along all segments of the viscera with long-lasting low-amplitude waves. The upper esophageal sphincter was normotonic and normo-functional; the pharyngeal pump was valid. The manometric picture was compatible with esophageal achalasia at an early stage.

The surgery of extramucosal cardio-myotomy sec. Heller and antireflux plastic acc. Thall in video-laparoscopy, was performed, with placement of nasogastric tube, removed on the first post-operative day.

**Fig. 6:**
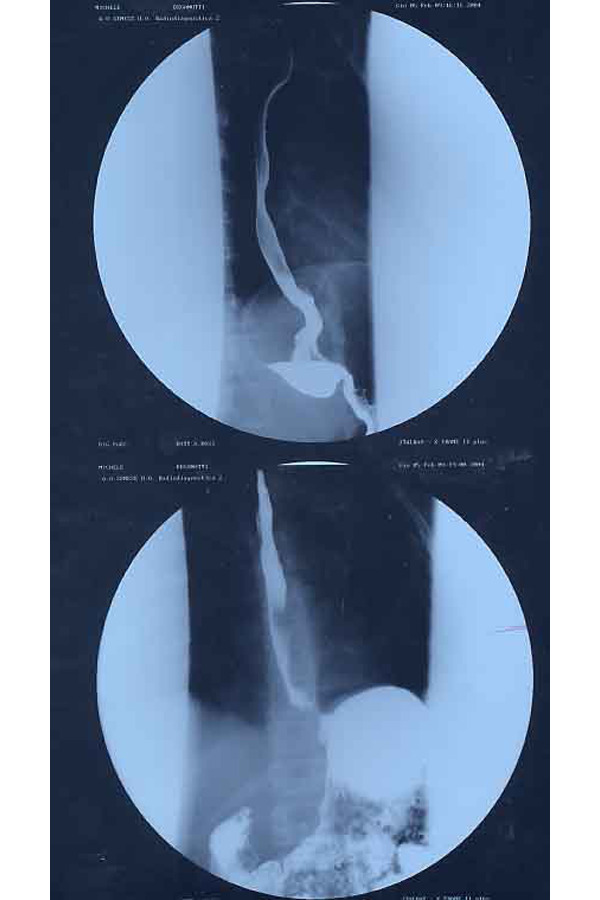
Post-operative image of LES

The postoperative course was normal, with discharge on the fifth day. The post-operative check was carried out three months after the surgery, the execution of upper digestive X-ray (Figure 6) highlighted: “Outcomes of surgery. The esophageal transit is normal without signs of gastro-oesophageal reflux. No gastric changes”. In addition, there was a weight increase of 3 kg.

## II. SURGICAL TECHNIQUE

The patient is positioned supine with legs spread and reverse Trendelemburg.

The first operator is positioned between patient’s legs, the cameraman on the patient’s right and the second operator on the left. Five trocars are positioned: on the left flank, on the hemiclavicular line (5 mm) for the retraction of the stomach during dissection, transumbilical for the optic (5 mm), left subcostal for liver retraction and left and right periumbilical for the operator. A pneumoperitoneum is created by insufflation of CO2 at 12 mmhg pressure.

After retracting the liver and stomach and identifying the gastro-oesophageal junction, the Phrenoesophageal ligament is dissected and the anterior isolation of the oesophagus is performed. Once isolated the viscera, myotomy is performed in order to cause the herniation of the mucosa; the muscular dissection extends for about 7cm. An intraoperative manometry confirms the reduction of pressure at the LES.

The operation is completed with the packaging of an anterior funduplication at 180 ° to protect the herniated mucosa and to prevent any gastro-oesophageal reflux.

The choice to perform a gastrostomy depends essentially on the patient’s clinical condition and his ability to eat properly. (5)

## III. DISCUSSION

The progressive loss of contractile LES activity results in a slowing of oesophageal clearance, with accumulation of food bolus. The entry of food into the stomach is facilitated by the hydrostatic pressure of the oesophageal content and by a contractile activity of the viscera which is particularly intense (vigorous achalasia-compensation phase) at the early stages of the disease (6).

The first symptoms may appear at this stage which, in the child, in most cases coincides with the school age. The alteration of the oesophageal transit is perceived as a vague and not well-defined sensation of bolus arrest at the retrosternal level identified by the term “dysphagia”. Initially, it is occasional, mild, common for both liquids and solids; often the child facilitates the feeding by performing repeated swallowing acts, eating in an upright position or holding his breath (7).

At the onset of the disease, dysphagia may be accompanied by episodes of intense spontaneous chest pain, caused by vigorous contractile activity of the oesophagus. The latter can push the contents both in the proximal and distal sense, causing regurgitation, halitosis, cough and pneumopathies ab ingestis. This picture generates in children fear to feed; they learn painfully to suppress appetite, becoming restless and malnourished (weight loss) (6).

In infants, the clinical picture may suggest the symptoms from gastro-oesophageal reflux, moreover much more frequent at that time of life, due to frequent regurgitation, apnea, inhalation of ingestions with pneumopathies and growth retardation (1).

The gold standard examination in the diagnosis of achalasia, is, without any doubt, the oesophageal transit with baritate meal which evidences, in most cases, an oesophageal dilatation with “mouse tail” narrowing at the LES level. The chest x-ray without contrast may show the absence of the gastric bubble, a hydroplane level above the cardiac shadow with mediastinum enlargement. (8)

In doubtful cases, manometry allows to evaluate the presence or absence of hypertone of LES and its incomplete or complete release after swallowing (9). Endoscopy allows to visualize the presence of plasters and to appreciate a characteristic shot when the instrument passes the cardias (sign of the “stream”) (10).

Treatment of achalasia is mainly surgical. In cases where the symptoms are particularly disabling, the surgery may be preceded by pharmacological therapy, consisting of calcium channel blockers or parasympathomimetic; the use of these drugs, resulting in transient relaxation of the muscles of the LES, has the effect of a temporary reduction of the disorders and it is reserved exclusively for adult patients (11).

The endoscopic dilatations, of a mechanical, hydrostatic or pneumatic nature (the most used), are effective, in paediatric age, in a percentage of cases of 46%; these techniques may, however, cause important complications, such as mucosal lacerations or increase the risk of perforations, either during endoscopic manoeuvres or during surgery. The current trend is, therefore, to prefer the surgical approach to endoscopic dilations (10).

The principal surgical technique is represented by modified extramucosal cardio-myotomy sec. Heller. Heller, in fact, described a surgical technique that involved two parallel myotomies, anterior and posterior; subsequently, Groeneveldt and Zaaijer modified this procedure with a single anterior longitudinal resection (12).

The surgical approach can be performed thoracically or abdominal. In any clinical case, we opted for the abdominal approach, because it allows to associate an antireflux plastic and is less traumatizing for the patient; moreover, we chose the video-laparoscopic technique, which further reduces surgical trauma (13).

The choice of performing anterior antireflux plastic (sec. Thall) guarantees a good protection of the herniated mucosa, avoiding subsequent gastro-oesophageal reflux problems; moreover, 180 ° anti-reflux plastics, at LES level, create a lower pressure increase compared to 360 ° plastics (eg Nissen), reducing the risk of secondary stenosis (14).

Possible complications of these types of surgery are intraoperative lacerations of the herniated esophageal mucosa or of the neighboring organs (e.g. liver and diaphragm) (15).

## IV. CONCLUSION

Extramucosal cardio-myotomy sec. Heller modified, completed by 180 ° anterior anti-reflux plastic (acc. Thall) represents the gold standard in the treatment of esophageal achalasia in pediatric age. In addition, video-laparoscopy reduces post-operative trauma, although it is closely linked to the operator’s ability, due to the difficulty of this technique (16).

As the analysis of our data showed, it is important to undertake a long-term follow-up, with clinical and radiological checks, aimed at promoting a gradual rebalancing of enteral nutrition and a good recovery of oesophageal function (17).
